# Protocol efficiently measuring the swelling rate of hydrogels

**DOI:** 10.1016/j.mex.2019.100779

**Published:** 2019-12-19

**Authors:** Katherine Zhang, Wuxiang Feng, Congrui Jin

**Affiliations:** a Department of Mechanical Engineering, Binghamton University, Binghamton, NY, 13902, USA; b Materials Science and Engineering Program, Binghamton University, Binghamton, NY, 13902, USA

**Keywords:** The proposed method is called sieve filtration method, Superabsorbent polymer, Swelling rate, Tea-bag method, Sieve method, Filtration method

## Abstract

Hydrogels are polymeric materials which can swell in water and retain a significant fraction of water within their structure without dissolving in water. Swelling rate is one of the most important properties of hydrogels. To measure the swelling rate, the profile of swelling capacity versus time of a hydrogel sample is obtained by performing free-absorbency capacity measurements at consecutive time intervals. Traditionally, either the tea-bag method, the sieve method, or the filtration method is used for the free-absorbency capacity measurements depending on the amount of the available sample and the desired precision. However, each method has its own systematic drawbacks. In this paper, a novel method called sieve filtration method is proposed for the measurement of the swelling rate of hydrogels. A protocol for this method is described in detail. The measurement results obtained from the proposed method and the traditional methods are compared. The proposed method has the following advantages over the traditional methods:

• It is more efficient than the traditional methods due to full contact of the hydrogel powders with water or aqueous solution as well as fast and complete removal of excessive fluid from the water-absorbed gel.
• It enables repeatable and reproducible measurement of the swelling rate of hydrogels.
• It is easy to implement, suitable for various types of hydrogels and aqueous solutions; and it requires small amounts of sample, minimal technical skill, and inexpensive equipment.

## Graphical abstract

**Figure:**
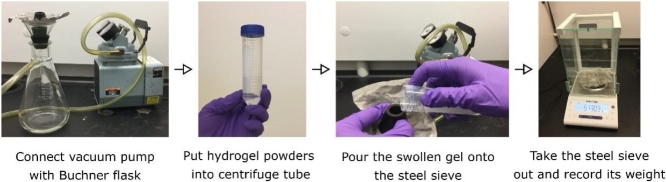

**Specification Table**

**Table:** 

Subject Area:	Engineering
More specific subject area:	polymer; superabsorbent polymer; hydrogels
Method name:	The proposed method is called sieve filtration method.
Name and reference of original method:	The traditional methods include tea-bag method; sieve method; and filtration method. The original method is described in the following two papers: http://doi.org/10.1617/s11527-018-1149-4 http://citeseerx.ist.psu.edu/viewdoc/download?doi=10.1.1.608.22&rep=rep1&type=pdf
Resource availability:	N/A

## Background

Hydrogels are polymeric materials which exhibit the ability of swelling in water and retaining a significant fraction of water within their structure without dissolving in water [1]. During the past decades, hydrogels have attracted enormous amounts of attention from the research community and the polymer industry owing to their wide range of applications in manufacturing contact lenses, hygiene products, tissue engineering, drug delivery systems, wound dressings, horticulture and agriculture, etc. [2].

**Fig. 1:**
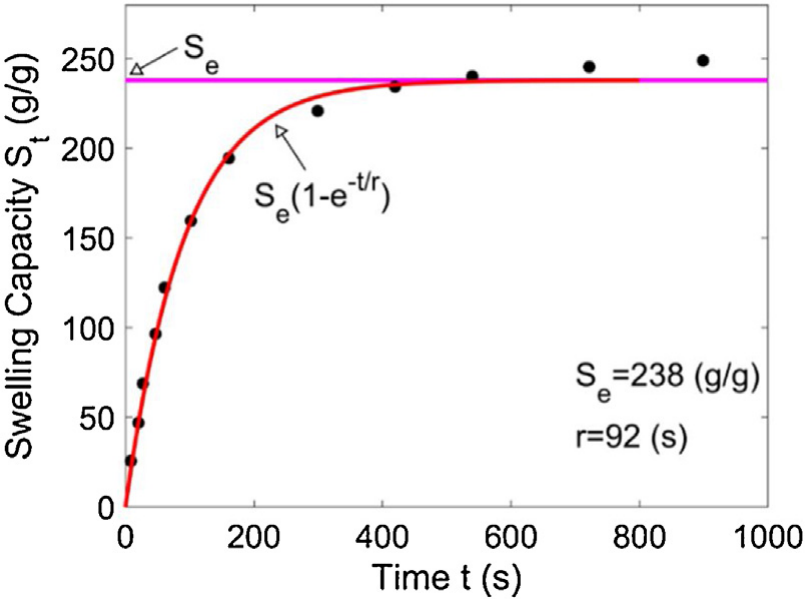
A typical profile of swelling capacity versus time of a hydrogel sample.

Swelling rate is one of the most important technical features of hydrogels. To measure the swelling rate, the profile of swelling capacity versus time of a hydrogel sample is often obtained by performing free-absorbency capacity measurements at consecutive time intervals, as shown in Fig. 1. The swelling values obtained from the measurements are then fitted into a Voigt model [3] as described by Eq. (1):

(1) $$S_{t}=S_{e}(1-e^{-t/r})$$

where t is the swelling time, $S_{t}$ is the swelling capacity at time t, $S_{e}$ is the equilibrium swelling, i.e., the swelling capacity at infinite time or the maximum water-holding capacity, and r is called the rate parameter, which is the time required to reach *0.63* of the equilibrium swelling.

Traditionally, either the tea-bag method, the sieve method, or the filtration method is used for the free-absorbency capacity measurements depending on the amount of the available sample and the desired precision. Each of the methods is briefly described below.

### Tea-bag method

The tea-bag method is the most conventional method for limited amounts of samples [[4], [5], [6], [7], [8]]. The initial weight of the powders $W_{0}$ is usually from 0.01 g to 0.03 g. The hydrogel powders are placed into a tea bag (acrylic/polyester gauze with fine meshes) and the bag is dipped in an excessive amount of water or aqueous solution for a prescribed period of time t. Then the tea-bag is placed on a dry cloth and gently wiped with another dry cloth to remove excessive fluid and weakly bound liquid. The tea bag is then weighed as $W_{2}$. The same procedure is also carried out for an empty bag, and the weight of the bag is measured as $W_{1}$. The swelling capacity at time t is calculated using Eq. (2):

(2) $$S_{t}=(W_{2}-W_{1}-W_{0})/W_{0}$$

### Sieve method

The sieve method is also called the rubbing method, which can be used to measure larger amounts of sample [9,10]. The initial weight of the powders $W_{0}$ is usually from 1.0 g to 2.0 g. The hydrogel powders are then poured into excessive amount of water or aqueous solution and dispersed with a stirring rod to ensure full contact with the liquid. After a prescribed period of time t, the water-absorbed gel is filtered through a 300-mesh wire gauze and the surface water is dried carefully by repeated rubbing under the gauze bottom using a piece of a soft open-cell polyurethane foam until the gel no longer slips from the sieve when held vertical. The weight of the sieve is measured as $W_{1}$. The water-absorbed gel together with the sieve is weighed as $W_{2}$. The swelling capacity at time t is calculated using Eq. (2).

### Filtration method

For the filtration method, the initial weight of the powders $W_{0}$ is typically from 0.01 g to 0.03 g. The procedure of this method has been previously documented in several publications [[11], [12], [13]]. A Buchner flask is connected to a vacuum pump, and then a funnel is firmly inserted into the flask. The measured hydrogel powders are put into a glass beaker, and an excessive amount of water or aqueous solution is then poured in. The hydrogel powders are dispersed with the stirring rod to ensure their full contact with the liquid. A pre-saturated filter paper with its weight recorded as $W_{1}$ is placed into the funnel. After a prescribed period of time t, the swollen sample is poured onto the center of the filter paper and the vacuum pump is turned on. After the excessive fluid is removed from the gel, the filter paper (with the gel on it) is taken out and its weight is recorded as $W_{2}$. Again, the swelling capacity at time t is calculated using Eqn. (2).

## The proposed method

Although the traditional methods have been widely used, they suffer from serious systematic drawbacks, as summarized in Table 1.

**Table 1:** Systematic drawbacks of the traditional methods.

	Tea-bag method	Sieve method	Filtration method	Proposed method
Problem 1: Can powders swell freely in the liquid?	✗	✓	✓	✓
Problem 3:Can residual inter-particle liquid be effectively removed?	✗	✗	✗	✓
Problem 4: Can water absorbed by the container be accurately determined?	✗	✓	✗	✓
Problem 2: How long is needed to remove excessive fluid (for 0.01 g sodium polyacrylate powders):	16 to 22 s	17 to 25 s	7 to 11 s	5 to 7 s

### Problem 1 – can powders swell freely in the liquid?

When the tea-bag method is used, the major issue is that the hydrogel powders are confined in a bag with limited room to move around. With such confinement, the hydrogel powders cannot swell freely in the liquid, and thus the liquid is not absorbed effectively by the powders in the interior areas where there is poor contact between the hydrogel powders and the liquid. Moreover, the swelling of the outer powders can hinder the liquid transport to the interior areas, leading to the measured swelling capacity significantly lower than their actual capacity. For example, according to Mechtcherine et al. [11], the absorption values in both cement filtrate solution and deionized water were systematically lower when the tea-bag method was used in comparison to those obtained using the filtration method. However, no quantification and scientifically sound proof of this critical aspect has been published up to date. Hence, in this study, the hypothesis will be quantitatively verified based on experimental results.

### Problem 2 – how long is needed to remove excessive fluid?

The sieve method is often used to address this drawback, i.e., using the sieve method, the hydrogel powders can swell freely in the liquid. However, this method is still far from being perfect, i.e., the excessive fluid is removed from the water-absorbed gel by repeated rubbing the gauze bottom using a piece of a soft polyurethane foam, which usually takes an extensive amount of time, resulting in inaccurate measurements. During the tests, to measure the value of $S_{t}$, the hydrogel powders are dipped in water or aqueous solution for a period of time t, and then the weight of the water-absorbed gel is measured after the excessive fluid is removed from the gel. Assuming that it takes Δt to remove the excessive fluid, the measured swelling capacity is actually $S_{t+\Delta t}$ instead of $S_{t}$, since the hydrogel powders continue absorbing liquid during the time of excessive fluid removal. When Δt is not negligible, the measured $S_{t}$ will be significantly higher than the actual capacity. In this study, this postulation will be quantitatively verified using the experimental results. Note that when the tea-bag method is used, the time to remove the excessive fluid is only slightly shorter than that used in the sieve method, so the tea-bag method also suffers from this issue.

### Problem 3 – can residual inter-particle liquid be effectively removed?

When the filtration method is used, the removal of excessive fluid from the water-absorbed gel is relatively fast, but not complete. The fine mesh of the filter paper can be easily clogged by the gel, preventing the residual inter-particle liquid from being effectively removed, which makes the measured value of $S_{t}$ much higher than the actual capacity. This assumption will be tested based on experimental measurements. In fact, this is a common issue among all the traditional methods. When the tea-bag method or the sieve method is used, only the excessive fluid on the surface is effectively removed, the residual inter-particle liquid may remain in the samples.

### Problem 4 – can water absorbed by the container be accurately determined?

When the filtration method or the tea-bag method is used, it is difficult to accurately determine the mass of water absorbed by the tea-bag or the filter paper. The absorption value of one piece of tea-bag or filter paper is typically estimated by averaging additional measurements on ten pieces of tea-bags or filter papers, but it may still deviate from the actual value.

### Other issues

There exists no detailed protocol for any of the traditional methods, and thus the accuracy of each method highly depends on individual behavior or laboratory common practice. Although some of the traditional methods are described in several internationally renowned specifications [[14], [15], [16], [17]], both the repeatability and the reproducibility have been recently questioned [11]. For example, when the tea-bag method is used, we found that a vigorous shaking of the bag during the time it is dipped in water or aqueous solution can significantly improve the contact between the hydrogel powders and the liquid, making the measured swelling capacity dramatically increased. Therefore, there is an urgent need for a detailed protocol enabling repeatable and reproducible measurement of the swelling rate of hydrogels.

Note that, besides the above-mentioned methods, the conductance method [18], the picture analysis method [19], and the calorimetric method [20] are also often used to measure the swelling rate of hydrogels, but each of them has its own significant drawbacks. The conductance method is to measure the conductivity change of sodium chloride in the tested solution during the swelling process based on the assumption that the hydrogels do not absorb sodium chloride until their swelling ratio reaches a certain value. However, the sodium chloride can also be absorbed by the hydrogels although the absorption rate is much slower than that of water, which makes the calculation of water absorption very complicated, requiring additional assumptions and calibrations [21]. For the picture analysis method, the hydrogel particles are put on a slide glass of a microscope connected to a videotape recorder, and the whole process of volume change of the hydrogel particles is videotaped. The volume change is then calculated based on the diameter change. However, the application of the picture analysis method is limited to only spherical particles [22]. For the calorimetric method, the heat of the swelling process is measured by a twin isoperibol calorimeter. However, the energy change in the system is very small or nearly zero after a short-time exothermic process, and thus this method is not effective to be used for tracing the whole swelling process [21].

### The proposed sieve filtration method

To address the drawbacks of the traditional methods, a novel method called sieve filtration method is proposed to measure the swelling rate of hydrogels. As listed in Table 1, this method does not have the four systematic drawbacks of the traditional methods. It allows for full contact of the hydrogel powders with water or aqueous solution as well as fast and complete removal of excessive fluid from the water-absorbed gel. It combines the positive attributes of both the sieve method and the filtration method. As a result, it enables repeatable and reproducible measurement of the swelling rate of hydrogels, and the swelling values obtained from the proposed method fit best to the Voigt model, as shown in the next section. In addition, this method is easy to implement, suitable for various types of hydrogels and aqueous solutions, and it requires small amounts of hydrogel powders, i.e., 0.005 g to 0.03 g, minimal technical skill, and inexpensive equipment.

Two commercially available hydrogels with different chemical compositions were tested in terms of their kinetics of absorption in deionized water. The hydrogel samples used in this study were potassium polyacrylate powders called BountiGel-P purchased from mOasis (Union City, CA, USA) and sodium polyacrylate powders called YanXing SAP purchased from YanXing (Renqiu, Hebei, China). The hydrogel powders were tested in their as-received original state. The diameter of potassium polyacrylate particles is from 0.2 mm to 0.6 mm and the diameter of sodium polyacrylate particles is from 0.6 mm to 1.0 mm. A dummy test was performed to estimate the amount of hydrogel powders needed for testing, and the results showed that the amount of the potassium polyacrylate powders should be in the range of 0.01 g to 0.03 g and the amount of the sodium polyacrylate powders should be in the range of 0.005 g to 0.01 g. The amount of hydrogel powders needed for testing is determined by the actual absorption capacity. The amount cannot be too much – there should be an excess of liquid for the hydrogel powders to freely swell to full extent, and the water-absorbed gel should not be so large that the removal of excessive fluid takes a significant amount of time. The amount cannot be too little, either – the water-absorbed gel should be large enough to cover the meshes of the sieve right above the hole of the size 7 rubber stopper, so that the vacuum can be effectively created to suck the excessive fluid out.

## Method details

Materials

• Potassium polyacrylate powders (BountiGel-P from mOasis, Union City, CA, USA)
• Sodium polyacrylate powders (YanXing SAP from Yanxing, Renqiu, Hebei, China)
• Deionized water
• Falcon 50 mL conical-bottom centrifuge tubes (Fisher Scientific, Pittsburgh, PA, USA)
• 300 mesh 10 cm × 10 cm 316 stainless steel woven wire sieve cloth
• Kimax1000 mL Buchner flask (Fisher Scientific, Pittsburgh, PA, USA)
• 75 mm diameter glass funnel (Fisher Scientific, Pittsburgh, PA, USA)
• Buchner funnel rubber stopper size 7 (Fisher Scientific, Pittsburgh, PA, USA)
• Neoprene filter adapter size 3 (Fisher Scientific, Pittsburgh, PA, USA)
• Neoprene filter adapter size 4 (Fisher Scientific, Pittsburgh, PA, USA)
• Fisherbrand stainless steel micro-spatula (Fisher Scientific, Pittsburgh, PA, USA)
• Fisherbrand traceable three-line alarm timer (Fisher Scientific, Pittsburgh, PA, USA)

Equipment

• Gast standard DOA-P504-BN diaphragm vacuum pump (Cole-Parmer, Vernon Hills, IL, USA)
• Mettler Toledo ML204 220 g ×0.1 mg analytical balance (Cole-Parmer, Vernon Hills, IL, USA)
• Benchmark BenchRocker 3D variable speed rocker (Sigma-Aldrich, St. Louis, MO, USA)

Procedure

**Fig. 2:**
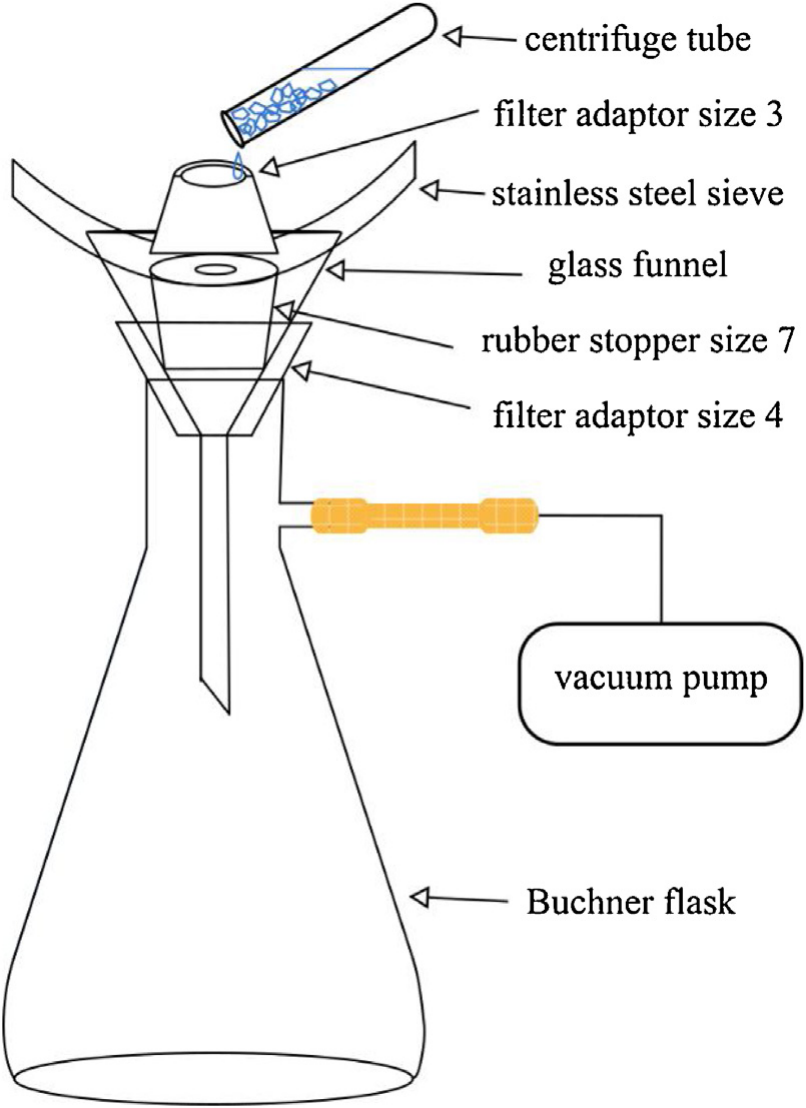
Schematics of the experimental setup used for the proposed sieve filtration method.

**Fig. 3:**
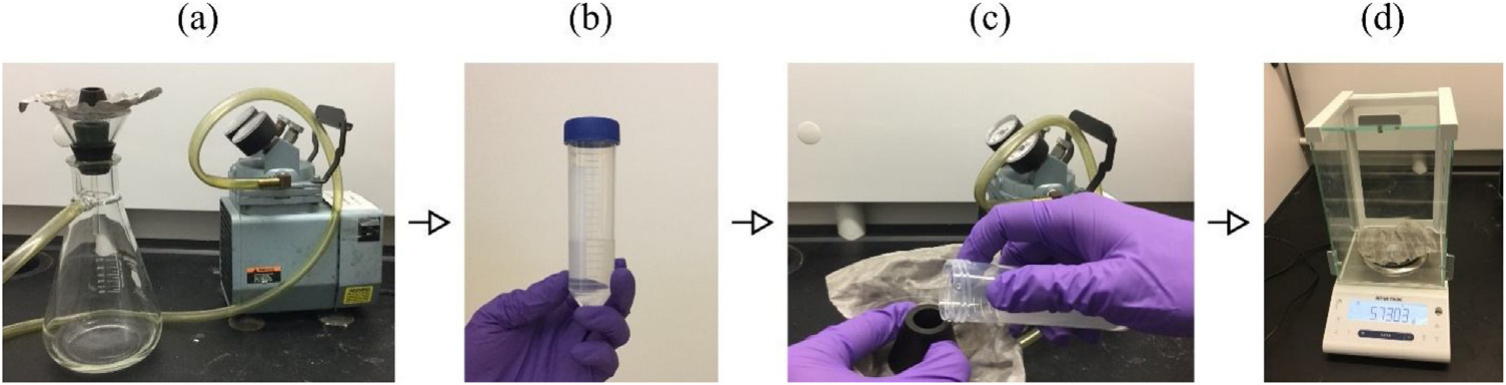
Procedures of using the proposed sieve filtration method for the measurement of the swelling rate of hydrogels: (a) connect the vacuum pump with the Buchner flask; (b) put the measured hydrogel powders into the centrifuge tube; (c) pour the swollen sample onto the steel sieve through the filter adaptor; and (d) take the steel sieve out and record its weight.
2 Using the micro-spatula, put approximately 0.02 g potassium polyacrylate powders or 0.01 g sodium polyacrylate powders on the analytical balance and record its weight as $W_{0}$. Put the measured hydrogel powders into the centrifuge tube, which contains 20 mL deionized water. Close the tube to minimize evaporation. Shake the tube by hand to improve contact between the hydrogel powders and the water, as shown in Fig. 3(b). If the prescribed period of time t is longer than 3 min, a rocker can be used to ensure full contact between the hydrogel powders and the water. It is important to note that conical-bottom centrifuge tube should be used to ensure that all the water-absorbed gels are transferred from the centrifuge tube to the mesh completely. Since the water-absorbed gels are very sticky and tend to get stuck at the corner, flat-bottom containers are not recommended.
3 After a prescribed period of time t, turn on the vacuum pump. Pour the swollen sample onto the steel sieve through the hole of filter adaptor size 3. After the excessive water is removed from the gel, turn off the vacuum pump, as shown in Fig. 3(c). The mesh of the sieve is much larger than that of the filter paper, allowing the residual inter-particles liquid to be effectively removed.
4 Take the sieve (with the gel on it) out and record its weight as $W_{2}$, as shown in Fig. 3(d).
5 The swelling capacity for the hydrogel at time t is calculated using Eqn. (2).

1 The weight of the steel sieve is measured as $W_{1}$. Assemble the two filter adaptors, the steel sieve, the glass funnel, and the rubber stopper on the Buchner flask, as shown in Fig. 2. Connect the Buchner flask to the vacuum pump, as shown in Fig. 3(a).

## Comparison with traditional methods

**Fig. 4:**
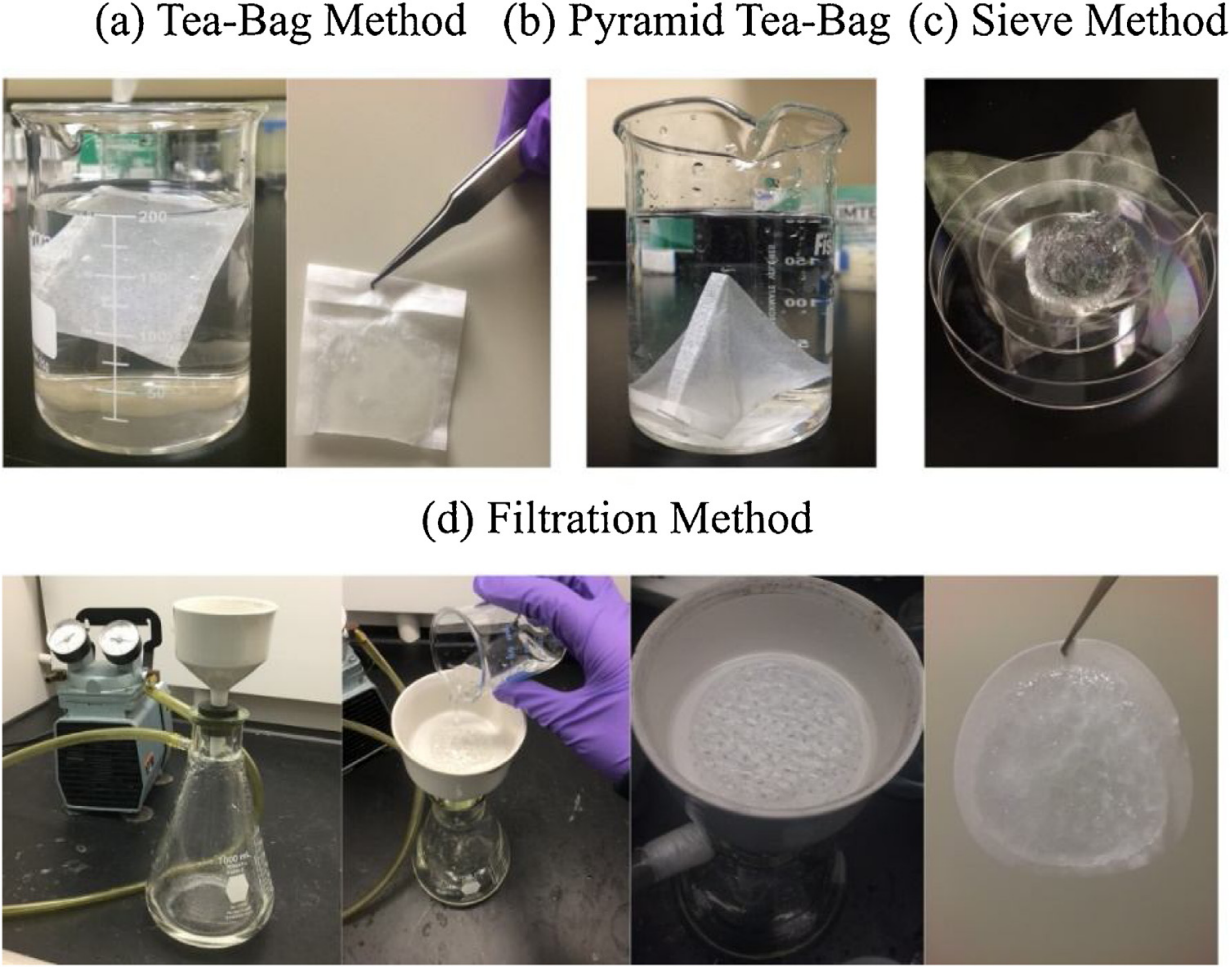
For the purpose of comparison, the swelling rate of the hydrogel sample is measured by the proposed sieve filtration method and the traditional methods including (a) the tea-bag method; (b) the pyramid tea-bag method; (c) the sieve method; and (d) the filtration method, respectively.

For the purpose of comparison, the profile of swelling capacity versus time of both the potassium polyacrylate powders and the sodium polyacrylate powders in deionized water is measured by the tea-bag method, the sieve method, the filtration method, and the proposed sieve filtration method, respectively. The results are discussed and compared. To check if the tea-bag method leads to poor contact between the liquid and the hydrogel powders in the interior areas, a variation of the tea-bag method is used by folding the tea-bags into pyramid shapes. Using the pyramid tea-bag method, the hydrogel powders have moderately increased room to swell freely inside the tea bag. If the swelling capacity measured by the pyramid tea-bag method is consistently higher than that obtained by the traditional tea-bag method, it shows that the inaccuracy of the tea-bag method is caused by the poor contact problem. The experimental setup used for each method is shown in Fig. 4. Each measurement was performed using 0.02 g potassium polyacrylate powders or 0.01 g sodium polyacrylate powders in triplicate and the hydrogels were not re-used.

**Fig. 5:**
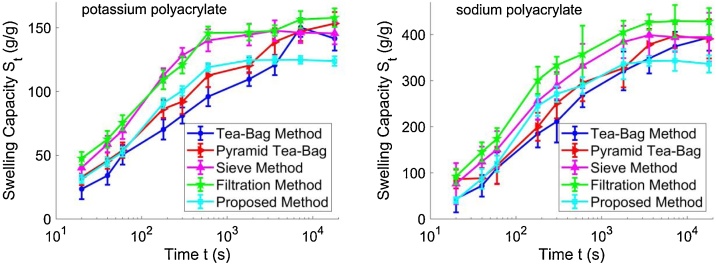
The profile of swelling capacity versus time of the hydrogel powders in deionized water obtained by the proposed sieve filtration method and the traditional methods, respectively. In each case, the swelling capacity is measured after 20 s, 40 s, 1 min, 3 min, 5 min, 10 min, 30 min, 1 h, 2 h, and 3 h. The error bar represents the standard deviation based on at least nine different measurements performed by three different experimenters.

The measured results are shown in Fig. 5. It can be seen that, within one hour, the swelling capacity measured by the tea-bag method is always significantly lower than the results obtained using the other methods. The swelling capacity measured by the pyramid tea-bag method is moderately higher than that obtained by the traditional tea-bag method, indicating that the inaccuracy of the tea-bag method is caused by the poor contact problem. However, the swelling capacity measured by the pyramid tea-bag method is still lower than the results obtained by the filtration method, the sieve method, or the proposed method, since the hydrogel powders are still confined in a bag and thus cannot swell completely freely in the liquid. After one hour, the swelling capacity measured by the tea-bag method or the pyramid tea-bag method becomes higher than that obtained by the proposed method, indicating that the liquid absorption by the powders even in the interior areas of the tea-bag is improved when the dipping time is long enough. Since the tea-bag method only effectively removes the excessive fluid on the surface and the residual inter-particle liquid remains in the samples, the equilibrium swelling capacity measured by the tea-bag method is higher than that obtained by the proposed method.

It also shows that the swelling capacity measured by the sieve method or the filtration method is always significantly higher than that obtained by the proposed method. When the sieve method is used, the removal of excessive water from the water-absorbed gel takes an extensive amount of time and the hydrogel powders continue absorbing water during the time of excessive fluid removal, making the measured swelling capacity significantly higher than the actual capacity. In addition, rubbing the gauze bottom cannot completely remove the excessive water, which further increases the measured value of swelling capacity. When the filtration method is used, the fine mesh of the filter paper can be easily clogged by the gel, preventing the residual inter-particle liquid from being effectively removed, which makes the measured swelling capacity much higher than the actual capacity.

The error bar represents the standard deviation based on at least nine different measurements performed by three different experimenters. It can be seen that the error bar for the proposed method is the smallest among the error bars for all the five methods, indicating that the proposed method has better repeatability and reproducibility over the traditional methods.

**Fig. 6:**
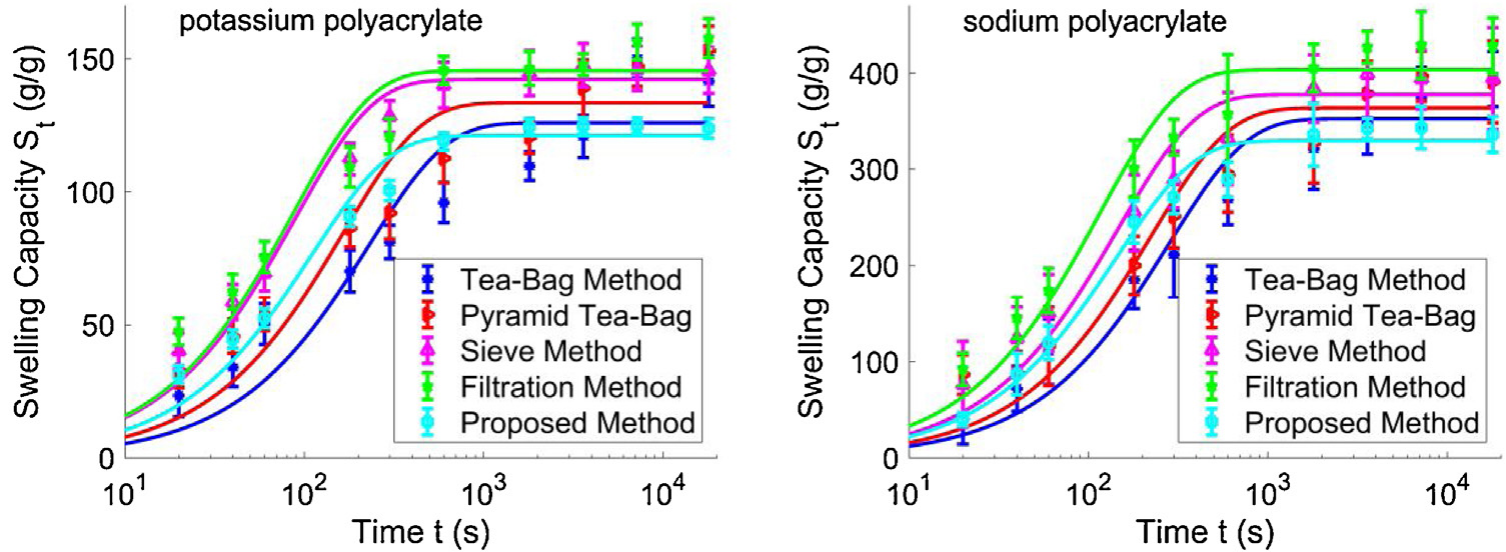
The swelling values obtained from the measurements are then fitted into the Voigt model.

The swelling values obtained from the measurements are then fitted into the Voigt model as described in Eq. (1). The fitted curves are shown in Fig. 6. The values of $S_{e}$, i.e., the equilibrium swelling, and r, i.e., the rate parameter, obtained using the five different methods are listed in Table 2. Also listed in Table 2 is the value of the mean squared error (MSE) of each fitted curve. It can be seen that the MSE value obtained from the proposed method is the smallest among the five MSE values for both the potassium polyacrylate powders and the sodium polyacrylate powders, indicating that the swelling values obtained from the proposed method fit best to the Voigt model.

**Table 2:** The values of the equilibrium swelling $S_{e}$, the rate parameter r, and the mean squared error MSE obtained using the five different methods for the potassium polyacrylate powders and the sodium polyacrylate powders, respectively.

Hydrogel Sample	Parameters	Tea-Bag	Pyramid Tea-Bag	Sieve	Filtration	Proposed Method
Potassium polyacrylate	r (s)	227.9	164.4	88.8	87.1	111.0
	$S_{e}$ (g/g)	126.0	133.6	142.2	145.6	121.2
	MSE	944.7	1156.3	757.3	811.9	243.7
Sodium polyacrylate	r (s)	281.5	222.4	147.1	116.5	143.8
	$S_{e}$ (g/g)	352.5	363.7	377.6	403.4	329.6
	MSE	276.4	250.9	50.2	155.2	49.2

## Additional information

To remove the excessive water faster, the proposed sieve filtration method can be further improved by using a larger funnel, a ribbed funnel, a funnel with larger surface area, a size 7 rubber stopper with enlarged hole, or a more powerful vacuum pump to accelerate the rate of filtration.

Note that the efficiency of the proposed method is closely related to the particle size of the hydrogel powders, the type of liquid used, the mesh width of the sieve, the pressure of the vacuum applied, and the vacuum time, etc. A thorough study needs to be carried out to investigate these relationships. This endeavor will be left as our future work.
